# Properties of Arsenic–Doped ZnTe Thin Films as a Back Contact for CdTe Solar Cells

**DOI:** 10.3390/ma12223706

**Published:** 2019-11-10

**Authors:** Ochai Oklobia, Giray Kartopu, Stuart J. C. Irvine

**Affiliations:** Centre for Solar Energy Research, College of Engineering, Swansea University, OpTIC Centre, St. Asaph Business Park LL17 0JD, UK; giray.kartopu@swansea.ac.uk (G.K.); s.j.c.irvine@swansea.ac.uk (S.J.C.I.)

**Keywords:** ZnTe:As back contact, CdTe, thin films, solar cells, metalorganic chemical vapor deposition (MOCVD)

## Abstract

As-doped polycrystalline ZnTe layers grown by metalorganic chemical vapor deposition (MOCVD) have been investigated as a back contact for CdTe solar cells. While undoped ZnTe films were essentially insulating, the doped layers showed significant rise in conductivity with increasing As concentration. High p-type carrier densities up 4.5 × 10^18^ cm^−3^ was measured by the Hall-effect in heavily doped ZnTe:As films, displaying electrical properties comparable to epitaxial ZnTe single crystalline thin films in the literature. Device incorporation with as-deposited ZnTe:As yielded lower photovoltaic (PV) performance compared to reference devices, due to losses in the open-circuit potential (V_OC_) and fill factor (FF) related to reducing p-type doping density (*N*_A_) in the absorber layer. Some minor recovery observed in absorber doping following a Cl-free post–ZnTe:As deposition anneal in hydrogen at 420 °C contributed to a slight improvement in V_OC_ and *N*_A_, highlighting the significance of back contact activation. A mild CdCl_2_ activation process on the ZnTe:As back contact layer via a sacrificial CdS cap layer has been assessed to suppress Zn losses, which occur in the case of standard CdCl_2_ anneal treatments (CHT) via formation of volatile ZnCl_2_. The CdS sacrificial cap was effective in minimising the Zn loss. Compared to untreated and non-capped, mild CHT processed ZnTe:As back contacted devices, mild CHT with a CdS barrier showed the highest recovery in absorber doping and an ~10 mV gain in V_OC,_ with the best cell efficiency approaching the baseline devices.

## 1. Introduction

Record efficiencies as high as 22.1% have been reported for cadmium telluride (CdTe) solar cells [1], making it one of the fastest growing and promising thin film technologies. CdTe continues to attract increasing interest in academia and industry as it is ideal for very high performance photovoltaic (PV) applications, mainly due to a high absorption coefficient >10^4^ cm^−1^, a bandgap of 1.45 eV and low-cost manufacturing attributes. Even with such reported high efficiencies, much work is still needed to close the gap towards the theoretical maximum of 30% [2]. 

Insertion of a wide band gap material between the CdTe absorber and metal contact has demonstrated promise for efficiency improvement [3]. This approach overcomes the problem of the Schottky barrier formed at CdTe/metal interface, for available metal contacts [4]. Another approach is incorporating a p+ region near the CdTe back surface [5]. In the former; the use of p-type ZnTe thin film is considered to be a good example. With a zincblende (cubic) structure and a bandgap of ~2.26 eV, ZnTe has negligible valence band discontinuity with respect to CdTe (which would not impede hole transport), and a large conduction band offset, which can be beneficial for electron back reflection to CdTe and hence minimize minority carrier recombination related losses at the interface to the metal back contact [3,6]. Additionally, very high p-type doping concentrations achievable with ZnTe can provide a low resistance Ohmic contact to the metal electrode. Cu-doped ZnTe back contact to CdTe have been credited with improvement in device efficiency [7], however Cu diffusion and subsequent long-term device stability remains an issue [8]. As an alternative to ZnTe:Cu, group V (N, As)-doped ZnTe can be used as a back contact. Specifically ZnTe:N has been recognised as a suitable back contact for CdTe thin film solar cells [9], also providing improved device stability, compared to ZnTe:Cu [10].

**Figure 1:**
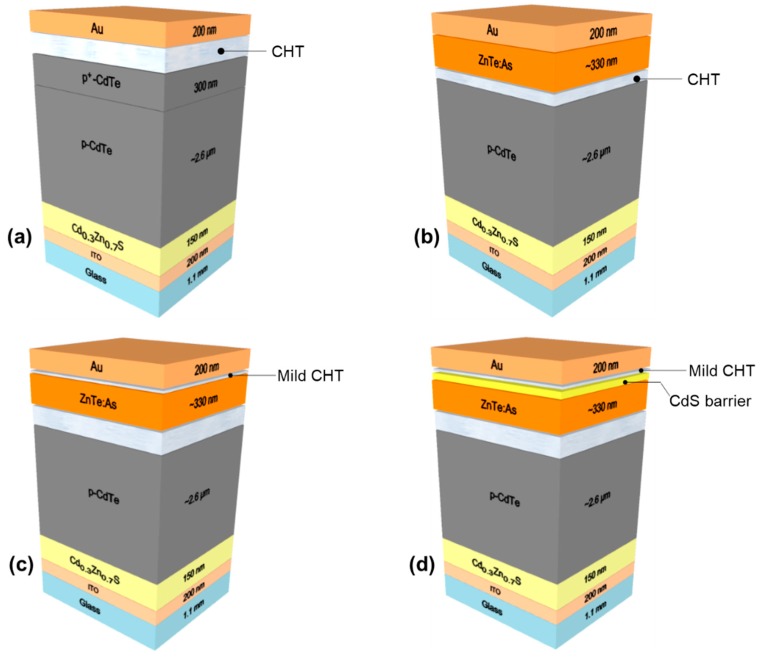
Schematic of (**a**) baseline CdTe cell; (**b**) ZnTe:As back-contacted CdTe cell, and (**c**) ZnTe:As back contacted cell with mild Cl-anneal without a CdS sacrificial layer (**d**) ZnTe:As back contacted cell with mild Cl-anneal using a CdS sacrificial layer (not to scale).

In our baseline CdTe thin film solar cell structure (Figure 1a), according to a superstrate configuration, a heavily As-doped (~10^19^ atoms cm^−3^) CdTe cap layer, denoted by the p+-CdTe back contact layer (BCL) was found to lower the device’s series resistance [5], resulting in efficiencies as high as 13.0% being achieved. However, dopability with CdTe is challenging, as a recent study by Kartopu et al [11] has shown As doping of polycrystalline CdTe with an upper limit of ~3 × 10^16^ cm^−3^. Nevertheless, p-type doping of ZnTe to very high levels is achievable in comparison, where doping concentrations as high as 1 × 10^18^ cm^−3^ for in-situ doped ZnTe:As have been reported [12,13]. With such doping levels, p-ZnTe BCL is expected to further reduce back contact resistance, and therefore contribute to improving the device’s series resistance. In this study, we sought to substitute the heavily doped (p+-CdTe) cap layer with a p+-ZnTe BCL, which has comparably higher doping efficiency, by using metalorganic chemical vapor deposition (MOCVD) to grow As-doped ZnTe with good conductivity. We investigated properties of the ZnTe:As BCL to CdTe thin film solar cells and reported post-deposition treatments, for cell performance improvement. Activation of the ZnTe BCL using conventional CdCl_2_ deposition followed by heat treatment (CHT) is known to be challenging due to Zn loss via the formation of highly volatile ZnCl_2_ during the process [14,15]. Therefore we also investigated and report on the development of an activation process by assessing a thin layer of MOCVD grown sacrificial CdS layer on ZnTe:As to control the BCL activation step. In addition to a slight performance improvement, we also showed evidence of the barrier function of CdS in minimizing Zn loss.

## 2. Materials and Methods 

Solar cells in this study were fabricated in the superstrate configuration in a horizontal atmospheric pressure (AP)-MOCVD reactor (Cryogenic Vacuum Technology, Milton Keynes, UK), using hydrogen as a carrier gas. The CdTe baseline cell structure was fabricated by depositing a window layer (Cd_1-x_Zn_x_S) on indium tin oxide (ITO)-coated boro-aluminosilicate glass substrates (4–8 Ω/□), followed by the CdTe absorber, comprising a ~2.6 µm As-doped p-CdTe layer terminated with a more heavily doped, p+-CdTe (~300 nm) back contact layer. Further experimental details can be found elsewhere [16]. Cell activation was achieved via CHT, performed by depositing a thick layer (~3.0 µm) of CdCl_2_ thin film at 200 °C, then anneal for 10 min at 420 °C under hydrogen ambient. After cooling to room temperature, the structure was taken out of the reactor and excess CdCl_2_ rinsed with deionized (DI) water, and a secondary post-deposition annealing was carried out at 170 °C, for 90 min in air ambient. Finally, the solar cell was completed by evaporation of Au metal back contacts through a shadow mask. Dimethylcadmium (DMCd), diisopropyltelluride (DIPTe), diethylzinc (DEZn), and tertiarybutylchloride (tBuCl) were used as the metalorganic precursors for Cd, Te, Zn, and Cl, respectively, whilst tris(dimethylamino)arsenic (tDMAAs) was used for the As dopant. The schematic layout of the baseline cell and ZnTe-contacted structures in this work are illustrated in Figure 1.

Before incorporating ZnTe:As BCL in the CdTe cell structure, reference single layers of the ZnTe thin films were first grown at 370 °C on uncoated boro-aluminosilicate glass substrates using MOCVD, whilst controlling its p-type property by varying the tDMAAs flows between 0 and 10 sccm, corresponding to partial pressures of 0–4.73 × 10^−6^ atm. The thicknesses of the reference thin films was measured to be ~500 nm. Transmittance and Hall-effect measurements were carried out to determine the optical and electronic properties of the corresponding ZnTe:As thin films. The heavily doped ZnTe achieved with 10 sccm of tDMAAs flow was subsequently incorporated into the p-CdTe absorber, as the back contact layer. Prior to ZnTe:As BCL deposition on to the p-CdTe back surface, the absorber base structure was activated similarly to the baseline structure, cooled to room temperature, excess CdCl_2_ rinsed off with DI water and returned to the MOCVD reactor within 24 h. Figure 1b shows the cell structure grown by omitting the p+-CdTe layer and then finished with ZnTe:As deposition. To improve ZnTe:As BCL crystallinity, annealing under flowing H_2_ gas at 420 °C for 10 min (i.e., Cl-free H_2_ anneal), was performed subsequently. In determining the optimal annealing conditions for the ZnTe:As BCL, the Cl-free H_2_ anneals were performed on different Glass/ITO/Cd_1-x_Zn_x_S/p-CdTe-CHT/ZnTe:As samples for times ranging between 0 and 30 min (at 420 °C), and also at temperatures ranging from 420 to 450 °C (for 10 min).

To assess the ZnTe:As back contact on p-CdTe thin film solar cell performance, a control device (i.e., a Glass/ITO/Cd_1-x_Zn_x_S/p-CdTe-CHT structure, without the p+-CdTe BCL), in addition to our baseline cell structure, was also fabricated. 

For the ZnTe:As BCL activation, we first performed a wet chloride treatment, by immersing a Glass/ITO/Cd_1-x_Zn_x_S/p-CdTe-CHT/ZnTe:As sample in a 10% ZnCl_2_/methanol solution for 2 min, left it to dry in air before transferring into the MOCVD reactor for a subsequent H_2_ anneal. The ZnCl_2_ wet treatment here was assessed as an alternative to our standard CHT treatment (for CdTe absorbers) and according to literature is expected to minimise or avoid Zn loss from the ZnTe:As BCL [14]. On another batch of Glass/ITO/Cd_1-x_Zn_x_S/p-CdTe-CHT/ZnTe:As samples, we also investigated the development of a relatively mild CHT activation step, for the ZnTe:As BCL, using a thin CdS cap layer [15] to minimise Zn loss during the process. After ZnTe:As BCL deposition on p-CdTe in the MOCVD reactor, and performing a Cl-free H_2_ anneal, the CdS (~80 nm) cap layer and a relatively thin layer of CdCl_2_ were sequentially grown. The resulting structure (Glass/ITO/Cd_1-x_Zn_x_S/p-CdTe-CHT/ZnTe:As/CdS/CdCl_2_) was then annealed at 420 °C for only 3 min in H_2_. Upon cooling down to room temperature, samples were removed from the MOCVD reactor, with excess CdCl_2_ rinsed off and the CdS layer selectively removed by etching in a 15% aqueous HCl acid solution and rinsed again with DI water to remove any residual products. Three different samples were processed: a reference (without the mild CHT or CdS layer, i.e. Glass/ITO/Cd_1-x_Zn_x_S/p-CdTe-CHT/ZnTe:As) and two other samples with the mild CHT but with and without a CdS sacrificial layer (Glass/ITO/Cd_1-x_Zn_x_S/p-CdTe-CHT/ZnTe:As/CdS-mild CHT and Glass/ITO/Cd_1-x_Zn_x_S/p-CdTe-CHT/ZnTe:As/-mild CHT). Prior to device completion by Au contact evaporation, all ZnTe:As back contacted device structures were subjected to a standard secondary annealing in ambient air at 170 °C for 90 min.

AM 1.5 J-V measurements were performed using an Abet Technologies Ltd. (Milford, CT, USA) solar simulator with the light power density calibrated using a GaAs reference cell. C-V characteristics were measured using a Solartron Impedance Analyzer (Ametek Scientific Instruments, Farnborough, UK). External quantum efficiency (EQE) measurements were carried out using a Bentham spectral response system (Bentham Instruments Ltd., Berkshire, UK). The system response was corrected by scanning the output of a c-Si reference detector. Energy dispersive X-ray spectroscopy (EDX), to determine elemental compositions, was carried out using a Hitachi TM3000 (Hitachi Ltd., Tokyo, Japan) table-top scanning electron microscope (SEM). X-ray diffraction (XRD) measurements were obtained from a D8 Discover (Bruker, Karlsruhe, Germany) diffractometer. Cl depth profiling was performed via secondary-ion mass spectroscopy (SIMS) with a Cameca IMS-4f instrument (LSA Ltd., Leicestershire, UK) with a Cs+ ion source operating with 10 keV energy and 20 nA current. Samples for SIMS were prepared by first cleaving ~1 × 1 cm^2^ specimens, and back surface subsequently etched in a 0.2% bromine solution in methanol (BrMeOH) for 5 s, rinsed with methanol and dried with a N_2_ gun. 

## 3. Results

### 3.1. Properties of As-Doped Polycrystalline (px) ZnTe Thin Films by MOCVD

Electrical properties of MOCVD grown reference px ZnTe thin films on boro-aluminosilicate glass substrate with different As flows, determined by Hall-effect measurements, are summarized in Table 1. Each thin film sample was annealed in air at 170 °C for 90 min prior to Hall-effect measurements, as this was found to be beneficial for improving the dopant activation [5]. The bandgap values in Table 1 were estimated from Tauc plots.

**Figure 2:**
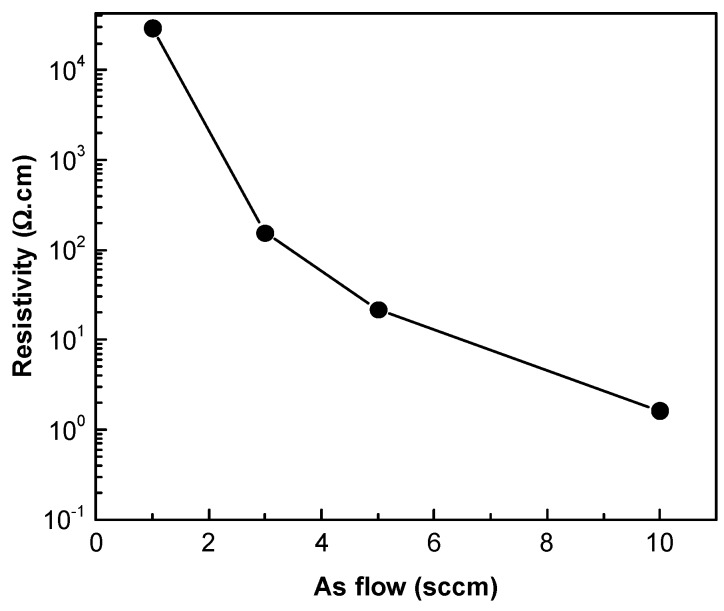
Resistivity change in As-doped px ZnTe reference thin films with As precursor flows.

Due to the highly resistive nature of the undoped ZnTe film, no reliable data was obtained by the Hall-effect measurements. When an As flow of 3 sccm was used during the ZnTe growth, a p-type conductive film with a bulk concentration of 5.33 × 10^17^ cm^−3^ was obtained. The resistivity however was noted to be high. Bulk concentration increases with increasing As flow to 4.50 × 10^18^ cm^−3^ for 10 sccm, with a corresponding decrease in the resistivity (Figure 2). Such low resistivities with high doping densities are expected to be beneficial to forming low–resistance Ohmic contacts in CdTe thin film solar cells. Becker et al. successfully demonstrated the utilization of a 20 nm ZnTe:As (epitaxial) MOCVD thin film, displaying 2 × 10^18^ cm^−3^ p-type carrier density, as the hole collecting back contact within a single crystalline CdTe/CdMgTe double heterostructure [17].

Optical properties investigated by measuring the transmittance spectra (see Supplementary Materials Figure S1) of the reference sample px ZnTe thin films showed absorption increase with As flow. The thin films were deposited to ~500 nm thickness. The corresponding optical bandgaps estimated from Tauc plots (Table 1) indicated a bandgap value of 2.23 eV for the undoped px ZnTe and this decreased slightly to 2.13 eV with the highest As doping used (10 sccm). 

To probe the impact of As doping on the microstructure, we compared the X-ray diffraction pattern of undoped and highly doped px ZnTe (see Figure S2). Three peaks can be seen at 2θ angles of 25.3°, 49.5°, and 52.0°, corresponding to preferential (111) and comparatively weaker (311) and (222) planes, in the undoped ZnTe thin film [3,8,14]. The (111) peak intensity was observed to reduce with doping (i.e., with 10 sccm of As) and the ZnTe (222) was absent, along with the emergence of additional peaks, indicating some disorder forming. The new peak at 38.3° corresponds to Te (102), suggesting As substitution in Te sites and in general recrystallisation of the ZnTe, whilst the other peaks at 29.6°, 44.1°, and 64.4° (denoted by symbol ▼) does not appear to correspond to any ZnTe phases. No shift in the (111) diffraction was noticed, which contrasts with reported XRD data for ZnTe:N [18], where a peak shift in (111) was induced by nitrogen doping, attributed to the embedding of nitrogen atoms in interstitial sites, resulting in films with decreased lattice parameter due to compressive stress. 

### 3.2. Device Incorporation of ZnTe:As to CdTe Thin Film Solar Cells

**Figure 3:**
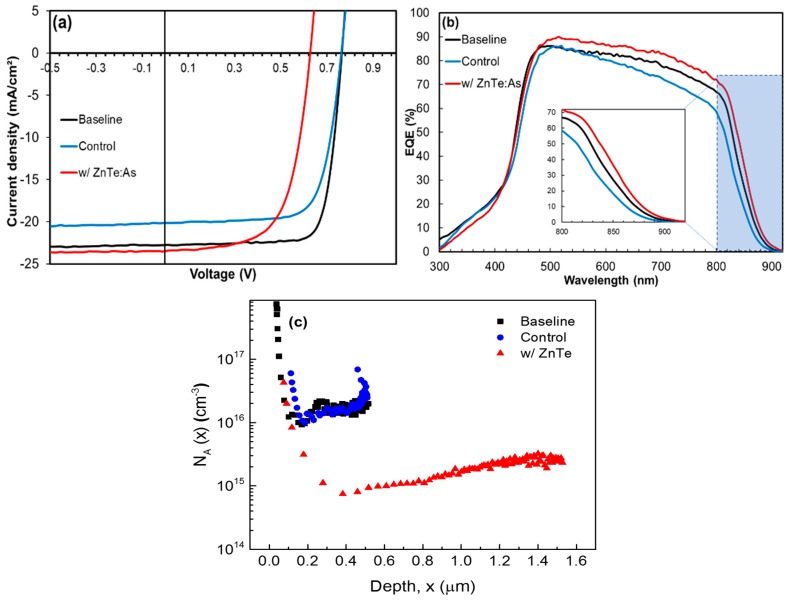
(**a**) Illuminated J-V curves (**b**) external quantum efficiency (EQE) spectra and (**c**) acceptor concentration profiles for: baseline, control, and ZnTe:As (with NO post-deposition treatment) back contacted devices.

A comparison of the illuminated J-V curves of as-deposited ZnTe:As back contacted CdTe thin film solar cell, baseline and control devices are shown in Figure 3a. The growth parameters for the ZnTe:As BCL incorporated into the p-CdTe structure were chosen based on the property results from reference ZnTe:As thin films (see Table 1). Dopant precursor flow was kept fixed at 10 sccm and the BCL film thickness was about ~330 nm (Figure 1b). Note that cells defined by the Au contacts on the ZnTe:As back contacted devices had to be isolated by scribing before J-V measurements, as ZnTe:As BCL was found to exhibit high lateral conductivity which contributed to current collection beyond the actual defined cell area.

From Figure 3a, open-circuit voltage (V_OC_), short circuit current density (J_SC_), and fill factor (FF) of 0.748 V, 20.2 mA/cm^2^, and 73% were measured in the control device respectively, corresponding to a best conversion efficiency (*ƞ*) of 11.10%. Whereas in the baseline cell structure, i.e., a control cell structure terminated with a p+-CdTe (~300 nm) BCL, the V_OC_, FF and J_SC_ all increased to 0.765 V, 22.8 mA/cm^2^ and 78%, respectively, yielding a *η* of 13.60%. By incorporating the p+-CdTe layer, *η* increased by 2.5%, confirming previous reports on the benefits of having a heavily As-doped CdTe BCL in the device structure [5]. However, for the cell with an as-deposited ZnTe:As BCL, a lower *η* of 9.30% was measured, due to the relatively low V_OC_ and FF (0.627 V and 64%), although having a slightly improved J_SC_ of 23.5 mA/cm^2^. The improvements in J_SC_ observed in both baseline and ZnTe:As contacted CdTe cells was found to be consistent with improved red responses evident in the EQE spectra (Figure 3b). Device series resistance (R_S_), on the other hand, improved in baseline and ZnTe:As contacted devices compared to the control sample, possibly indicating the contribution of a heavily doped BCL in reducing the contact resistance to the Au metal contact [6]. Comparing the baseline and ZnTe:As contacted devices, the improvement in R_S_ is slightly better in the former, despite the higher conductivity of ZnTe:As, which is expected to yield even lower overall series resistance with the ZnTe:As back contacted devices. The as-deposited ZnTe:As here was not subjected to any post-treatment prior to Au contact application, unlike the p+-CdTe in the baseline structure which received the activation anneals along with the CdTe absorber. This could probably be the reason for the slightly higher Rs values observed for the ZnTe:As BCL device. Therefore an appropriate post deposition treatment for the ZnTe:As BCL in the CdTe solar cell structure is expected to further improve device performance. Shunt resistances, R_SH_ greater than 1000 Ω⋅cm^2^ were measured for all three devices, which, together with small R_S_, contributed to enhancing the devices’ FFs, except for the device with ZnTe:As BCL. Figure 3c shows the acceptor concentration (*N*_A_) profiles determined from C-V measurements for all three devices. As is evident, *N*_A_ for both control and baseline devices are comparable (~1.34 × 10^16^ and 1.0 × 10^16^ cm^−3^, respectively), with corresponding narrow depletion widths, whilst for devices with as-deposited ZnTe:As BCL, *N*_A_ degrades contrastingly by about one order of magnitude (~1 × 10^15^ cm^−3^) with a relatively widened depletion region. These effects seem to suggest that the incorporation of an as-deposited ZnTe BCL to the CdTe cell structure can actually lower the active carrier densities within the junction (depleted) and bulk (non-depleted) regions of the CdTe cell. Comparing the illuminated J-V parameters and *N*_A_ profiles, a correlation can be noted between *N*_A_ values and the respective V_OC_s measured for the three device samples. This relationship is consistent with what is generally accepted in the literature, that is, carrier concentration significantly impacts (proportionally) the device V_OC_ [19]. Widening of the depletion region, on the other hand, is expected to increase the minority carrier transport to the junction over a larger depth of the absorber, thereby increasing the Jsc and device red response, as experimentally observed.

### 3.3. Optimisation of ZnTe:As BCL to CdTe Thin Film Solar Cells

#### Cl-Free H_2_ Annealing

**Figure 4:**
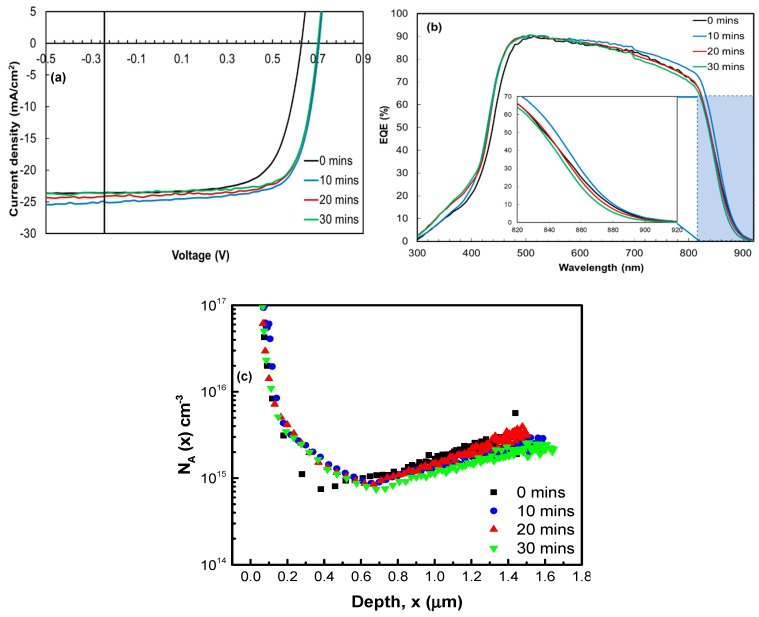
(**a**) Illuminated J-V curves (**b**) EQE spectra and (**c**) acceptor concentration profiles of ZnTe:As back contacted devices annealed at 420 °C for different times.

In order to investigate activation of the ZnTe:As BCL, we performed a series of post-deposition Cl-free H_2_ anneals on a batch of Glass/ITO/Cd_1-x_Zn_x_S/p-CdTe-CHT/ZnTe:As samples. First, we kept the annealing temperature fixed at 420 °C (i.e., the same annealing temperature for the baseline device activation) and annealed the samples for times ranging from 0 to 30 min. Corresponding illuminated J-V curves, EQE spectra and *N*_A_ profiles of ZnTe:As back contacted devices, as a function of the H_2_ annealing times, are summarized in Figure 4. 

Note that parameters of the device with as-deposited ZnTe:As BCL (see Section 3.2 and Figure 3) is denoted as having received 0 min of annealing in Figure 4, which is considered as the reference device here. Following the Cl-free H_2_ anneal for 10 min, we see an improvement in the power conversion efficiency (9.30% to 12.00%) primarily due the enhancements in V_OC_ (0.627 to 0.705 V) and FF (64% to 69%) whilst J_SC_ remained relatively the same. As for R_S_, we also note that by performing the Cl-free H_2_ anneal, there was a slight improvement in the series resistance compared to our baseline device mentioned in Section 3.2. The significant (2.7%) efficiency improvement here underscores the significance of ZnTe:As BCL activation and that it appears to be critical to high device performance. For the other samples annealed beyond 10 min (20 and 30 min), no further improvements in FF or V_OC_ were seen, instead J_SC_ began to degrade slightly, resulting in conversion efficiencies around 11.70%. The loss in J_SC_ was found to correlate well with the EQE spectra (Figure 4b); for longer annealing times above 10 min, the red response slightly degrades. According to the calculated R_SH_ and J_0_ (reverse saturation current density) parameters as a function of annealing time (Table S1), no correlation between the V_OC_ and these parameters can be seen. The bulk acceptor concentration in Figure 4c does not appear to improve after annealing for 10 min, but rather remains relatively unchanged with a slight indication of the onset of degradation at higher annealing times (i.e., 30 min). Although cell efficiency was enhanced as a result of the Cl-free H_2_ annealing, extended annealing times greater than 10 min was not found to be effective in producing any further improvement in the device performance; rather, this was detrimental particularly to the J_SC_. 

**Figure 5:**
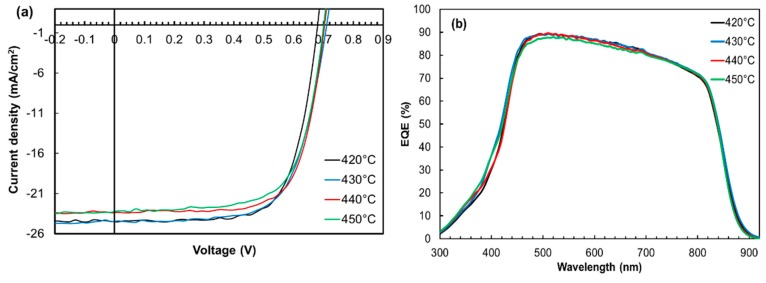

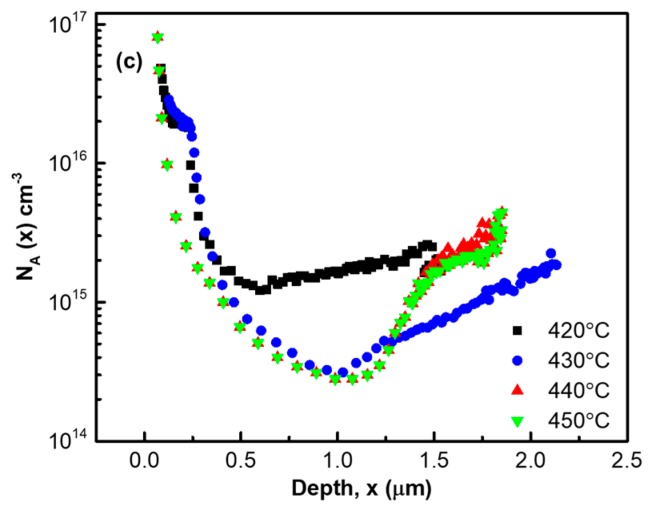
(**a**) Illuminated J-V curves (**b**) EQE spectra and (**c**) acceptor concentration profiles of ZnTe:As back contacted devices annealed at 420, 430, 440, and 450 °C for 10 min each.

Next, we optimised the Cl-free H_2_ annealing temperature by performing a series of ZnTe:As BCL post-deposition Cl-free H_2_ anneals on another batch of samples at higher temperatures; 430, 440, and 450 °C in addition to the 420 °C (reference), for a fixed time of 10 min (optimum time chosen from the data in Figure 4). Figure 5 shows the illuminated J-V curves, EQE spectra, and *N*_A_ profiles of ZnTe:As back contacted CdTe cells following Cl-free H_2_ anneal at different temperatures. As seen in Figure 5a, devices annealed at 430 °C yielded similar J_SC_ (24.5 mA/cm^2^) as those on which a 420 °C anneal was performed, but deteriorated by about 2–2.3 mA/cm^2^ for higher temperature treatments, i.e., 440 and 450 °C. In general, increasing the annealing temperature beyond 420 to 430 °C for samples in this work did not result in improved device performance, rather the J_SC_ was found to degrade. The bulk acceptor concentration was particularly negatively impacted (Figure 5c) as it reduced with annealing temperature beyond 420 °C. In view of this, we expected a corresponding loss in the respective cell’s V_OC_s, according to previous observations, but this was not the case here. The reason for the absence of a correlation between V_OC_ and *N*_A_ for the different annealing temperatures here is unclear at this stage. Based on the *N*_A_ profiles (Figure 5c), 420 °C was chosen as the suitable Cl-free H_2_ annealing temperature.

Meanwhile, the XRD patterns of the ZnTe:As BCL on CdTe absorber after Cl-free H_2_ annealing at 420 and 450 °C (Figure S3) were obtained to elucidate the impact of annealing temperature on the microstructure of the ZnTe:As back contacted CdTe cells. The sample annealed at 420 °C showed the ZnTe preferential orientation of (111), as it is consistent with the diffraction peak in the reference ZnTe:As thin film. In the case of the 450 °C annealed sample, a slight shift in the (111) ZnTe diffraction peak towards (111) CdTe was seen, suggesting microstructural changes and change in ZnTe composition with Cd incorporation towards Zn rich CdZnTe. Comparing with surface SEM images (Figure S4), we observe some coalescence of distinct crystalline structures after annealing at 440 to 450 °C, suggesting that annealing at higher temperatures (>420 °C) is unlikely to be productive for high device performance.

Based on the observations from the foregoing; the Cl-free H_2_ anneal for 10 min at 420 °C is considered as the optimum ZnTe:As BCL post-deposition annealing condition in this work, thus these conditions were employed in all subsequent experiments.

### 3.4. ZnTe:As BCL Thickness Optimisation

We also examined the effect of ZnTe:As BCL thickness and the measured PV parameters of CdTe thin film solar cells with different ZnTe:As BCL thicknesses are summarized in Table 2. 

As seen in Table 2, the device performances are generally similar for BCL thicknesses between 100 and 330 nm. In the case of the 50 nm BCL, however, a significant drop in efficiency, primarily due to poor FF observed, caused by high R_S_, which may result from non-uniform and incomplete coverage of the ZnTe:As layer on the p-CdTe surface [3]. Therefore, to ensure that p-CdTe is completely covered by the ZnTe:As BCL film, the BCL thickness for all remaining samples were kept at nominally 330 nm. 

### 3.5. Cl-Annealing BCL Post-Deposition Treatments

**Figure 6:**
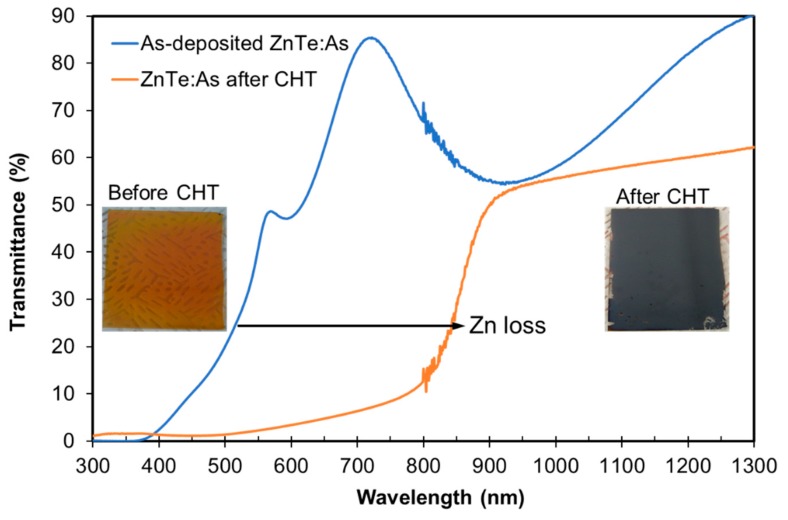
Transmittance spectra and image of a reference thin film of ZnTe:As before and after the standard CHT.

Effect of a standard CdCl_2_ heat treatment, normally applied to CdTe absorbers, is first checked on reference ZnTe:As thin films on glass substrate. The transmittance spectra of reference ZnTe:As thin films, with and without the standard CHT, are shown in Figure 6. Following the CHT, a shift in the absorption edge towards longer wavelengths, towards that of CdTe, indicative of Zn loss and formation of Cd-Te, was observed. 

The obvious Zn loss and formation of Cd–Te bonding are believed to be through the formation of highly volatile ZnCl_2_ during the CHT process [14,15], confirming the difficulty that is expected with performing the standard CHT device activation step following the growth of the ZnTe BCL on the CdTe absorber. This would eliminate the ZnTe phase from the BCL due to its full conversion to the CdTe phase. Thus, in order to suppress the Zn loss in the presence of Cd precursor, we first attempted a wet ZnCl_2_ treatment alternative [20], on a sample of as-deposited ZnTe:As BCL device structure, which resulted in rather poor device performance (results not shown). Thereafter, we also tried to apply the ZnCl_2_ thin film via MOCVD. However, this proved to be a challenging effort, as the growing ZnCl_2_ phase was majorly volatile and did not adhere well to the substrate as a thin film coating, thus unlikely to result in an effective Cl activation of the ZnTe:As BCL.

We then proceeded to assess a milder CHT process for the ZnTe BCL; employing, for the first time, a sacrificial CdS cap layer. This effort was motivated by the work of Shimpi et al [15], who carried out a mild CdCl_2_ heat treatment and partially activated their CdZnTe absorbers for thin film solar cells, utilising a thin CdS barrier layer which minimized Zn loss from the CdZnTe phase. The mild CHT approach with a CdS cap was first assessed using reference thin films of ZnTe:As on boro-aluminosilicate glass substrate. Transmittance spectrum of a CdS capped reference ZnTe:As thin film which received a mild CHT indicated a much reduced Zn loss, indicated by the smaller shift in the absorption edge compared to another film which received the standard CHT with and without the CdS cap (see Figure S5 for details). Under standard CHT conditions, the CdS cap was beneficial in retaining Zn, which was further improved by the mild CdCl_2_ treatment with a CdS cap. With this result in mind, we proceeded to evaluate this promising process on the Glass/ITO/Cd_1-x_Zn_x_S/p-CdTe-CHT/ZnTe:As device structure. We note that the CdS layer was removed by etching before the metallization of the back surface with Au contacts.

**Figure 7:**
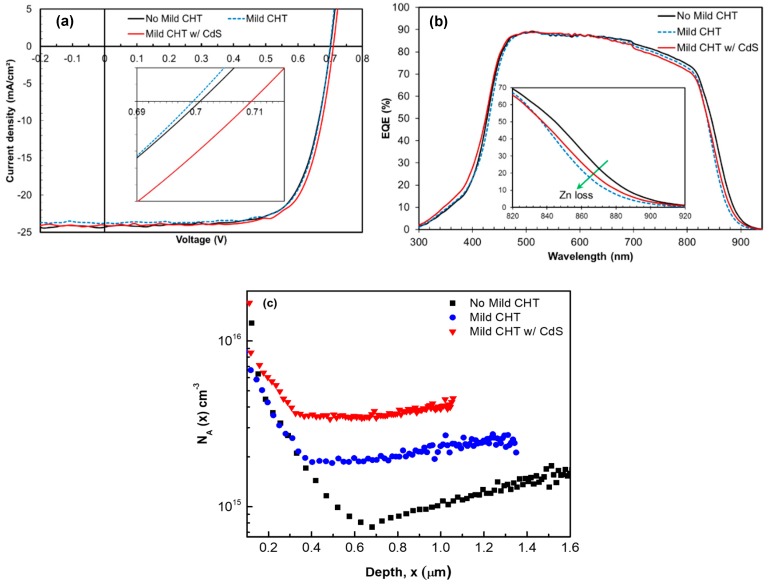
(**a**) Illuminated J-V curves (**b**) EQE spectra, and (**c**) acceptor concentration profiles of ZnTe:As back contacted devices: no mild treatment, with additional mild CHT (with and without CdS cap layer).

Figure 7 shows the illuminated J-V characteristics, EQE spectra, and *N*_A_ plots for mild CdCl_2_-annealed ZnTe:As BCL devices (both with and without the CdS cap layer), as well as a reference device without the additional mild CHT.

Device performances for all three samples are relatively similar, with the best conversion efficiency of ~12.3% from the device on which a mild CHT was performed on the ZnTe:As BCL with a sacrificial CdS barrier layer present. FF improves slightly from an average of 70.6% (no mild CHT) to 72.5% and 72.13%, respectively, for mild CHT devices. However, a slight drop in J_SC_ is observed following the Cl-anneal treatments, with the lowest mean J_SC_ measured in the sample without a CdS barrier layer during treatment. From EQE spectra in Figure 7b, a good correlation between J_SC_ loss and a declined near-infrared response (~800–900 nm region) can be seen, which is attributable to changes in the original ZnTe:As BCL composition following the mild CHT step. Zn loss causes a decline in the near IR response. The sample with the poorest red response correlated well with the device having the lowest J_SC_. The changes in this case are particularly connected to Zn loss. A V_OC_ improvement of about 10 mV was also measured in the device which received mild CHT in the presence of a CdS barrier layer. Except for the uncapped CdCl_2_-annealed sample, which showed a rather slight loss in V_OC_, the V_OC_ improvement correlates with the *N*_A_ determined from C-V measurements (see Figure 7c), whereby the acceptor concentration is seen to improve from ~1 × 10^15^ cm^−3^ (no mild CHT) up to ~4 × 10^15^ cm^−3^ (mild CHT with CdS cap) with corresponding narrowing depletion widths. In Figure 7b, we observe a small increase in the slope of the EQE at long wavelengths for devices on which mild CHT was carried out. This feature has been previously reported for CdTe:As solar cells with varied As doping [11], to be associated with increasing *N*_A_, and is consistent with our measured acceptor concentration (see Figure 7c).

ZnTe:As BCL compositional analysis (EDX) shown in Table 3 indicate a 23% loss in the Zn content (at %) following the mild CHT without the CdS cap. But this was less in the case of the sample device on which mild CHT was performed with a CdS layer (12% Zn loss), with an accompanying increase in Cd at %.

**Figure 8:**
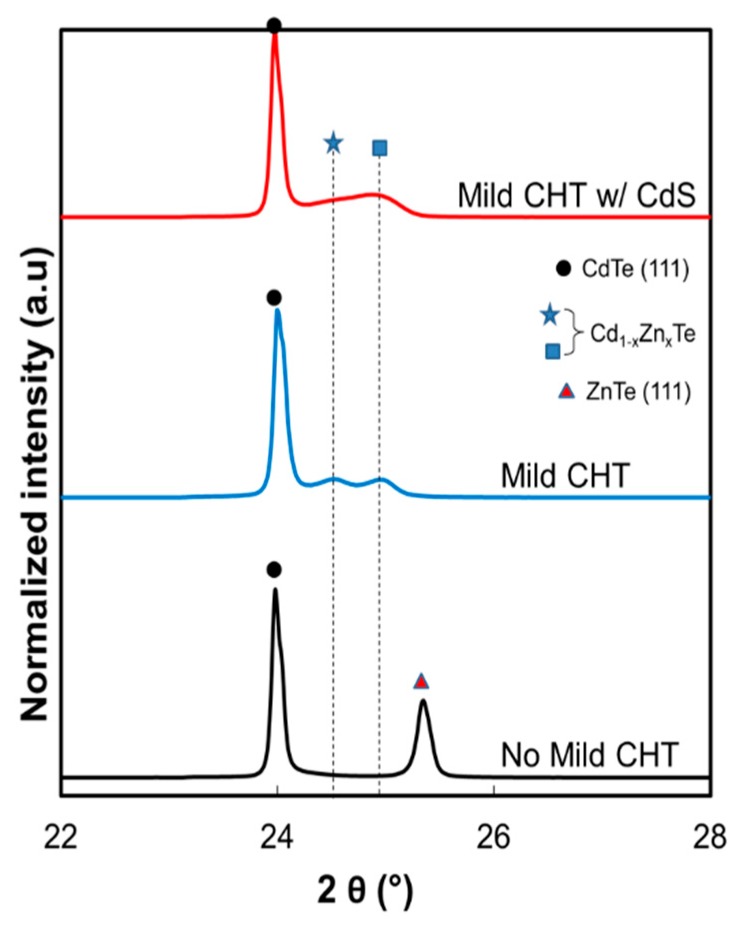
XRD patterns of ZnTe:As back contacted CdTe thin film solar cell structures.

Figure 8 shows the XRD patterns of ZnTe:As BCL on CdTe thin film structures for the three samples presented in Figure 7 and Table 3. Diffraction peaks close to CdTe (23.98°) [3] can be seen for all samples, which is likely an indication of the preferential orientation along the (111) plane of the underlying p-CdTe layer. The other peak at 25.34° for the sample without the mild CHT corresponds to the cubic ZnTe (111) reflection [3,18]. For the samples on which a mild CHT was performed (with and without a CdS cap), diffraction peaks can be seen between CdTe (111) and ZnTe (111), identified as belonging to the ternary Cd_1-x_Zn_x_Te (CZT) [21], which is consistent with the EDX analysis. The double CZT-like diffraction peaks seen in the uncapped mild CHT sample (24.52° and 24.92°) suggest a bi-layer of CZT structure formed with varying Zn compositions. From Vegard’s law it can be predicted that these layers have the compositions 44 and 74 at %, respectively. Note that composition cannot be determined accurately by EDX data (Table 3) due to interference of Te and Cd signals from the underlying CdTe absorber. For the CdS capped mild CHT cell, the CZT layer seems to have formed a graded composition with a dominant Zn-rich region.

**Figure 9:**
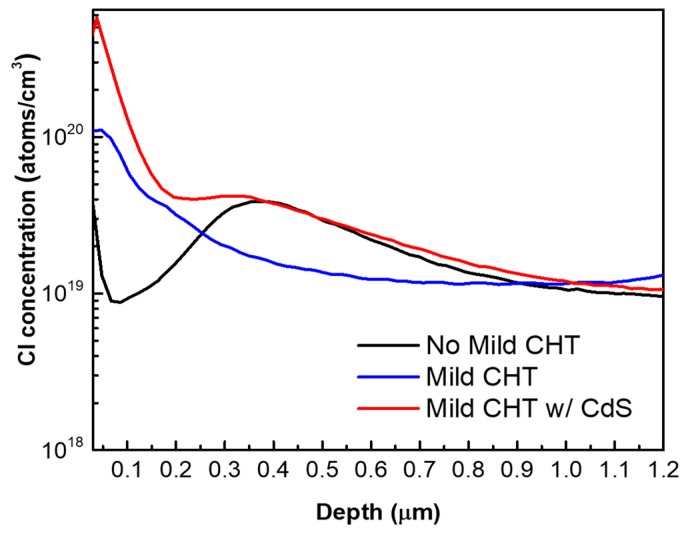
SIMS depth profile of Cl concentration for ZnTe:As BCL cells having received no mild CHT, mild CHT, and mild CHT with a CdS cap layer.

SIMS depth profiles of Cl in the device samples—no mild CHT, mild CHT (with and without a CdS barrier layer)—are presented in Figure 9. No Cl loss in the bulk p-CdTe absorber during subsequent ZnTe:As BCL deposition is observed. As expected, Cl incorporation in the ZnTe:As BCL can be seen following the mild CHT, but less so in the sample without a CdS cap. Cl accumulation can also be observed around the p-CdTe/ZnTe:As interface for the non-treated device, which is less prominent in the sample which had a CdS barrier during the mild CHT, and almost absent in the case of the uncapped, mild CHT sample.

## 4. Discussion

It is clear from Table 1 that reasonably high doping concentrations can be achieved in px ZnTe via in-situ As doping with MOVCD. Such bulk carrier concentrations demonstrated here for ZnTe:As, compares well with reported values for single crystal p-ZnTe [12,13]. Following device incorporation, the as-deposited ZnTe:As back contacted CdTe solar cell performed rather poorly compared to control and baseline device structures. Despite the J_SC_ enhancement (compared to control and baseline cells), the loss in the V_OC_ and FF was responsible for the poorer performance of the ZnTe:As BCL device. V_OC_ deterioration appeared to be correlated with an order of magnitude loss in absorber carrier density (*N*_A_). The sharp drop in *N*_A_ is believed to be the culprit, as it significantly impacts V_OC_ [19]. We are uncertain as to the origin of the decade loss in *N*_A_ following ZnTe:As BCL incorporation; however, a number of probable factors are worth taking into consideration. Interface contamination is one of such factors: following CdCl_2_ heat treatment post-deposition of a p-CdTe bulk absorber, the device structure was removed from the MOCVD reactor and excess Cl rinsed prior to reintroducing it back in the reactor for ZnTe:As BCL growth. The possible surface contamination acquired during the process may produce additional surface defects, and recombination sites, contributing to reduction in some device parameters [14]. Another important factor is the large lattice mismatch (~5.8%) between CdTe and ZnTe:As [3]. It is believed that this could lead to interface states and enhance recombination losses. If the minority carriers (electrons) are reflected back to CdTe from the energetically higher positioned ZnTe conduction band, these carriers can readily recombine via these interface states. 

By performing the Cl-free H_2_ anneal (420 °C, for 10 min) post ZnTe:As BCL deposition, minor recovery of *N*_A_ can be seen (Figure 4c). Although slight as it may appear, the physical origin of this thermally induced recovery is yet unknown. However, according to Wolden and co-workers [22], interface state passivation from interdiffusion at p-CdTe/ZnTe:As interfaces could partly be responsible for the observed recovery in the acceptor concentration. This phenomenon and other factors could also be at work in the case of the secondary mild CdCl_2_-anneal treatment post BCL deposition (with and without a CdS barrier layer; see Figure 7c). Additionally, the mild CHT induced conversion of ZnTe to Cd_1-x_Zn_x_Te compositions, inferred from transmittance and XRD results (Figure S5 and Figure 8), is expected to minimise the strain between the ZnTe BCL and the p-CdTe layer and improve the interface quality. This is expected because Cd_1-x_Zn_x_Te has a smaller lattice mismatch to CdTe compared to ZnTe [3]. Consequently, this could result in eliminating some interface defect states responsible for the degradation of *N*_A_ through recombination losses.

Note also that during the ZnTe:As BCL deposition at 370 °C and subsequent device anneals performed to improve the BCL, the CdTe absorber can be considered as undergoing additional post annealing which could restructure it by overtreatment. According to Abbas et al [23] post annealing of CdS/CdTe devices can result in Cl removal from absorber grain boundaries and the re-emergence of stacking faults, consequently detrimental to device performance. As discussed above (in relation to Figure 9) we do not see Cl loss from bulk or the back surface region of the absorber. Additionally, the performance was observed to be enhanced following the Cl-free H_2_ annealing as well as the mild CHT performed on our samples. Therefore, Cl loss related effects due to post annealing does not play a significant role for our devices.

## 5. Conclusions

In summary, we have investigated polycrystalline ZnTe:As back contacts to CdTe thin film solar cells grown by MOCVD. Highly conductive px ZnTe:As thin films with p-type carrier concentration as high as 4 × 10^18^ cm^−3^ by in-situ As doping, appropriate for back contact use in CdTe thin film solar cells, was achieved. Although, a slight improvement in J_SC_ was measured in as-deposited ZnTe:As back contacted devices, both V_OC_ and FF degraded, largely owing to a loss in absorber acceptor concentration (*N*_A_). A (Cl-free) H_2_ anneal carried out following ZnTe:As BCL deposition at optimum conditions (420 °C and 10 min) induced some minor recovery in *N*_A_ and V_OC_ and improvement to BCL crystallinity, indicating the need for a suitable ZnTe:As BCL post-deposition (activation) treatment, which is critical to device performance. When the standard (relatively aggressive) CdCl_2_ heat treatment, applied to activate the CdTe cell, was performed post ZnTe:As BCL deposition, then full conversion of ZnTe was observed into the CdTe phase due to the formation of volatile ZnCl_2_. 

A milder CdCl_2_-anneal treatment investigated using a thin CdS cap on the ZnTe surface (as a sacrificial barrier layer) caused an appreciable enhancement in the *N*_A_ and mitigated Zn loss. About 10 mV improvement in the V_OC_ was measured in devices which received the additional mild CHT with the CdS cap compared to untreated and non-capped mild CHT reference devices. Although some Zn loss was still noted with the use of a sacrificial barrier layer on the ZnTe surface, the concept showed promise towards full activation of the ZnTe:As BCL, without excessive Zn depletion and loss of *N*_A_. Mild CHT on the ZnTe film with a CdS cap was seen to be the most effective approach leading to *N*_A_ recovery in this study. Acceptor concentration recovery is considered to be largely due to p-CdTe/ZnTe:As interface improvement. Additionally, a slight reduction of lattice mismatch is implied from the resulting Cd_1-x_Zn_x_Te formation following mild CHT with a CdS cap. To further minimise Zn loss, other materials such as Al_2_O_3_ as an alternative to CdS [24] could be investigated. Additionally, direct deposition of the ternary alloy Cd_1-x_Zn_x_Te, which has an intermediate lattice mismatch (less than ZnTe) to CdTe, can be studied as an interlayer between p-CdTe and ZnTe:As.

## Figures and Tables

**Table 1:** Electrical parameters from Hall-effect measurements on ZnTe:As thin film samples deposited on uncoated glass substrate with different amounts of As.

As (sccm)	Bandgap (eV)	Bulk concentration (cm^−3^)	Mobility (cm^2^/V·s)	Resistivity (Ω·cm)	Conductivity (S/cm)
0	2.23	-	-	-	-
1	2.24	-	-	2.93 × 10^4^	3.41 × 10^−5^
3	2.21	5.33 × 10^17^	7.54 × 10^−2^	1.55 × 10^2^	6.45 × 10^−3^
5	2.20	1.11 × 10^18^	2.74 × 10^−1^	2.14 × 10^1^	4.68 × 10^−2^
10	2.19	4.50 × 10^18^	8.48 × 10^−1^	1.63 × 10^0^	6.14 × 10^−1^

**Table 2:** PV parameter summary of CdTe thin film solar cells with different ZnTe:As back contact layer thicknesses.

ZnTe:As (nm)	*η* (%)	J_SC_ (mA/cm^2^)	V_OC_ (V)	FF (%)	R_S_ (Ωcm^2^)	N_A_ (cm^−3^)	W_d_ (µm)
50	10.4	23.3	0.689	65	5.00	1.7 × 10^15^	1.01
100	11.9	23.7	0.696	72	1.45	1.9 × 10^15^	0.96
200	11.8	24.1	0.691	71	1.62	3.2 × 10^15^	0.78
330	11.7	24.1	0.701	69	1.69	2.0 × 10^15^	0.81

**Table 3:** Summary of EDX compositional data from ZnTe:As back contacted region of CdTe cells with and without additional mild CdCl_2_ heat treatment (CHT).

Element	No Mild CHT	Mild CHT	Mild CHT w/CdS
Zn (at %)	39.0	16.3	26.5
Cd (at %)	4.6	31.8	24.8
Te (at %)	56.4	51.9	48.8

## Supplementary Materials

The following are available online at https://www.mdpi.com/1996-1944/12/22/3706/s1, Figure S1: Transmittance spectra of ZnTe thin films with different amounts of As; Figure S2: Comparison between XRD patterns of ZnTe thin films on boro-aluminosilicate glass substrate; undoped (0 sccm) and doped with As (10 sccm); Figure S3: XRD patterns of ZnTe:As reference film, ZnTe:As back contacted CdTe thin films annealed at 420 °C and 450 °C; Figure S4: SEM of surface images of ZnTe:As BCL after different heat treatments; Figure S5: Transmittance spectra of reference thin films of as deposited ZnTe:As before and after standard CHT (with and without CdS sacrificial layer) and ZnTe:As after mild CHT with CdS sacrificial layer
