# Supplementary material for: Properties of Arsenic–Doped ZnTe Thin Films as a Back Contact for CdTe Solar Cells
Source: Materials (Basel). 2019 Nov 10;12(22):3706. doi: 10.3390/ma12223706 (PMC6888150; doi:10.3390/ma12223706)
Supplement: Supplementary file 1 [file materials-12-03706-s001.pdf]

materials

MDPI

Article

Properties of Arsenic–Doped ZnTe Thin Films as a Back Contact for CdTe Solar Cells

Ochai Oklobia *, Giray Kartopu and Stuart J. C. Irvine

Centre for Solar Energy Research, College of Engineering, Swansea University, OpTIC Centre, St. Asaph Business Park LL17 0JD, UK; giray.kartopu@swansea.ac.uk (G.K.); s.j.c.irvine@swansea.ac.uk (S.J.C.I.)

* Correspondence: ochai.oklobia@swansea.ac.uk

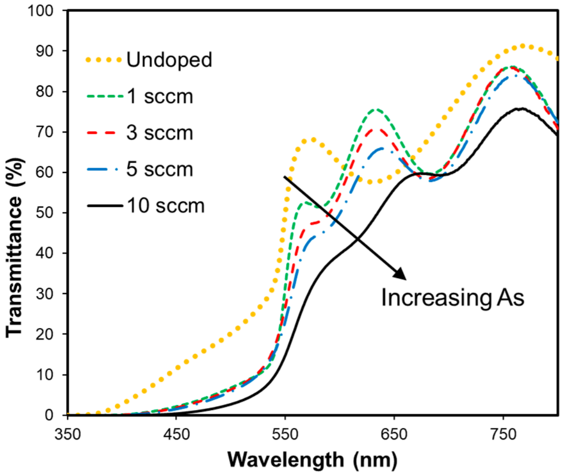

Figure S1. Transmittance spectra of ZnTe thin films (~500 nm) with different amounts of As.

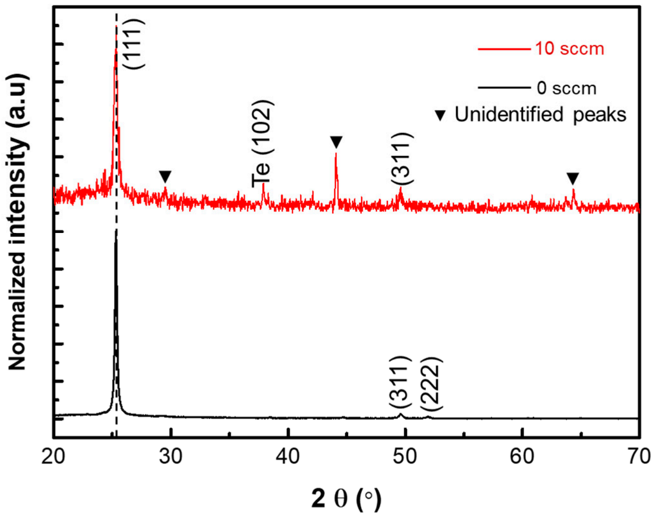

Figure S2. Comparison between XRD patterns of ZnTe thin films on boro-aluminosilicate glass substrate; undoped (0 sccm) and doped with As (10 sccm). Unidentified peaks are denoted by ▼.

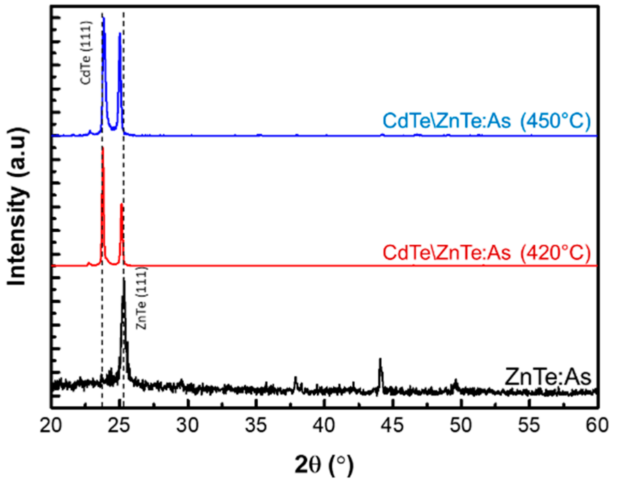

Figure S3. XRD patterns of ZnTe:As reference film, ZnTe:As back contacted CdTe cells annealed at 420 °C and 450 °C.

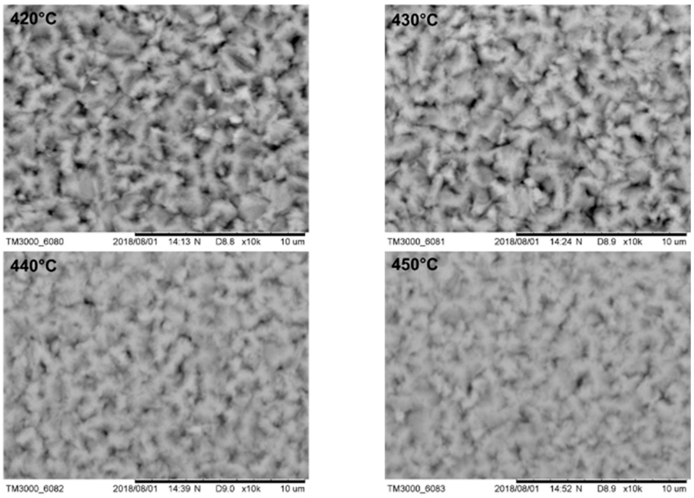

Figure S4. SEM of surface images of ZnTe:As BCL after different heat treatments.

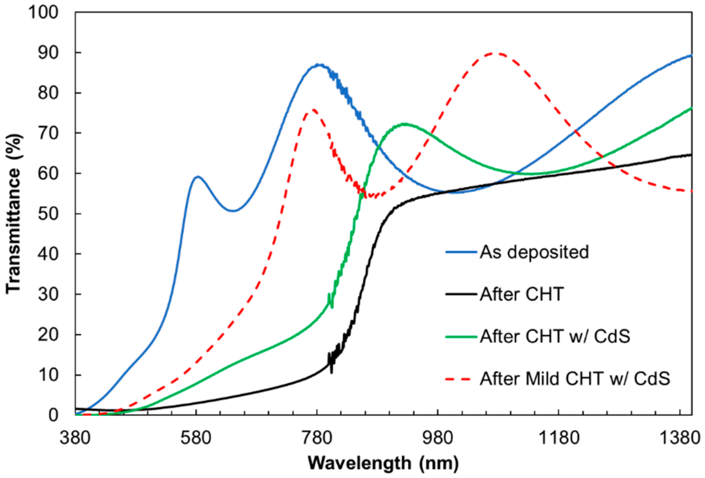

Figure S5. Transmittance spectra of reference thin films of as deposited ZnTe:As before and after standard CHT (with and without CdS sacrificial layer) and ZnTe:As after mild CHT with CdS sacrificial layer.

Table S1. Light shunt resistance (R_{SH}) and dark reverse saturation current density (J_0) as a function annealing time.

Cl-free H_2 annealing time (mins)	R_{SH} (Ω.cm^2)	J_0 (mA/cm^2)
0	2486.3	3.95×10^{-5}
10	695.0	1.54×10^{-5}
20	1239.2	1.53×10^{-5}
30	1379.9	6.11×10^{-5}

© 2019 by the authors. Submitted for possible open access publication under the terms and conditions of the Creative Commons Attribution (CC BY) license (http://creativecommons.org/licenses/by/4.0/).
